# Analysis of aroma‐active volatiles in an SDE extract of white tea

**DOI:** 10.1002/fsn3.1954

**Published:** 2021-01-09

**Authors:** Qi Lin, Hui Ni, Ling Wu, Shu Yi Weng, Lijun Li, Feng Chen

**Affiliations:** ^1^ College of Food and Bioengineering Jimei University Xiamen China; ^2^ Key Laboratory of Food Microbiology and Enzyme Engineering Technology Xiamen China; ^3^ Research Center of Food Biotechnology of Xiamen City Xiamen China; ^4^ DAMIN Foodstuff (Zhangzhou) Co., Ltd Zhangzhou China; ^5^ Department of Food, Nutrition and Packaging Sciences Clemson University Clemson SC USA

**Keywords:** aroma‐active volatiles, gas chromatography–mass spectrometry (GC‐MS), gas chromatography‐olfactometry (GC‐O), sensory evaluation, simultaneous distillation‐extraction (SDE), white tea

## Abstract

White tea is a famous Chinese tea that is cooked at boiling point before drinking. The simultaneous distillation‐extraction (SDE) was used to collect volatile compounds during tea cooking. The SDE extract was dominated with green, floral, roasted and woody notes, and weak sweet note. There were 32 volatile compounds identified via gas chromatography–mass spectrometry analysis, and 19 of them had strong fragrance based on the gas chromatography‐olfactometry analyzed results. Hexanal, 2‐hexenal, *cis*‐3‐hexen‐1‐ol, and camphene were the main contributors to the green note. The floral note was mainly contributed by 2‐hexanone, benzeneacetaldehyde, *trans*‐linalool oxide, and linalool, and the sweet note was induced by *trans*‐*β*‐damascenone. The roasted note was mainly contributed by 2‐pentyl‐furan. The woody note was mainly contributed by *trans‐α*‐ionone and *trans‐β*‐ionone. Four putative reaction pathways, including amino acid degradation, carotene degradation, Maillard reaction, and glycosides hydrolysis, were figured out to explain the generation of aromatic‐active volatiles at high temperatures. This study added our knowledge on tea aroma under cooking as well as other thermal treatments.

## Graphical abstract

Total 32 volatile compounds were identified via gas chromatography–mass spectrometry analysis. Among them, 19 volatile substances had significant fragrance based on gas chromatography‐olfactometry analysis. Hexanal, 2‐hexenal, *cis*‐3‐hexen‐1‐ol, and camphene contributed green note. 2‐Hexaone, benzeneacetaldehyde, *trans*‐linalool oxide, and linalool contributed floral note. *trans*‐*β*‐Damascenone contributed sweet note. 2‐Pentyl‐furan contributed roasted note. *trans‐α*‐Ionone and *trans‐β*‐ionone contributed woody note. Amino acid degradation, carotene degradation, Maillard reaction, and glycosides hydrolysis were figured out to explain the generation of aromatic‐active volatiles at high temperatures.

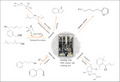

## 1 INTRODUCTION

Tea (*Camellia sinensis)* is one of the most important economical plants, and tea infusion is popularly consumed all over the world (Guo et al., 2018). Teas are mainly divided into six groups based on the fermentation degree and handling method, including green tea, white tea (WT), yellow tea, oolong tea, black tea, and dark tea (Yang et al., 2013). There are many functions of teas for our body, such as regulating intestinal flora (Liu et al., 2020), controlling body weight (Heber et al., 2014), and preventing cardiovascular diseases (Anandhan et al., 2013). Park et al. showed the extract from green tea could prevent cancer (Park et al., 2020). Ge et al. found antioxidant and bacteriostatic effects in yellow tea essential oil, for instance, hexanal possessed antioxidant potential and linalool had strong bacteriostatic effect (Ge et al., 2019).

Teas always have attractive and unique aroma that determined the quality of tea products. The aromas of teas are different according to the quality of its leaves, the process for making teas, and the environment for drinking tea. For example, the Japanese green tea Sencha was popularly consumed due to the pleasant green and floral notes (Tan et al., 2019). However, the unpleasant stale odor was found in the aged green tea that affected the quality of green tea (Dai et al., 2020). Xu et al. found that ripened Pu‐erh tea was dominated with floral, old and woody notes, and a raw Pu‐erh tea was dominated with floral and fruity notes (Xu et al., 2016). Sichuan Dark brick tea had a long‐lasting aged fragrance, while Sichuan Fuzhuan brick tea had a long‐lasting fungi floral note (Nie et al., 2019). The black tea steeped at 95°C had a more pleasant aroma with mild green, roasted, and fruity notes, the counterpart steeped at 80°C had a sweet fragrance with floral note, and those steeped at 60 and 70°C had more reinforced woody and fatty notes (Wang, Han, et al., 2019; Wang, Zeng, et al., 2019). Tao et al. reported that green tea infusion extracted at higher temperature had a stronger roasted note, while a lower‐temperature extraction resulted in a stronger floral note (Tao & Liu, 2019).

Volatiles that contributed to tea aromas could be extracted through the methods such as headspace solid phase microextraction (HS‐SPME; Xu et al., 2016), solvent‐assisted flavor evaporation (Sonmezdag et al., 2019), stir bar sorptive extraction (Trapp et al., 2018), simultaneous distillation‐extraction (SDE; Xu et al., 2016). Among them, SPME was more efficient for extracting low‐molecular weight compounds, while SDE was more appropriate for extracting compounds with high boiling point (Sheibani et al., 2016). Subsequently, the volatile compounds could be analyzed via gas chromatography–mass spectrometry (GC‐MS; Xu et al., 2016), gas chromatography‐olfactometry (GC‐O; Trapp et al., 2018), electronic nose (Rocchi et al., 2019), and gas chromatography–combustion–isotope ratio mass spectrometry (Sciarrone et al., 2018). By using these methods, more than 700 volatile compounds, including alcohols, esters, alkenes, ketones, and aldehydes were detected in various teas (Guo et al., 2018). A volatile compound could have a special odor; in addition, the volatile could affect tea aroma via interacting with another aromatic volatile (Mao et al., 2018).

**FIGURE 1:**
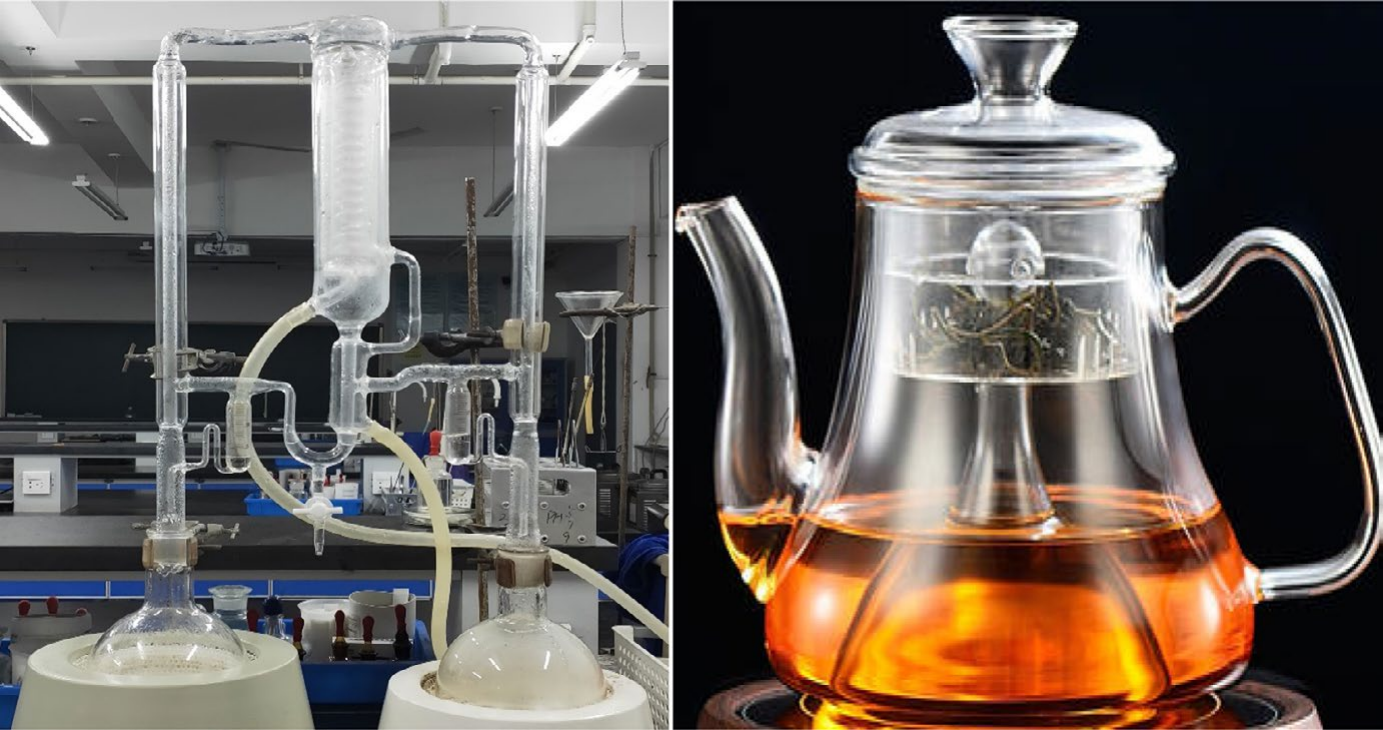
Similarity of simultaneous distillation‐extraction (SDE) instillation (left) and tea cooking pot (right). *Note:* The temperature for SDE is the same as that for tea cooking. The SDE instillation and tea cooking pot have similar size in the outlet

White tea, a famous Chinese tea, is prepared from young buds with silvery hairs via the combined processes of withering, roasting, and firing (Deng et al., 2017; Rusak et al., 2008). A study showed fresh tea leaves are withered at 30°C with a relative humidity of 47% for 36 hr, and then roasted at 120°C for 20 min followed by drying at 80°C for 20–30 min to a moisture content about 5% (Guo et al., 2018). White tea could help with lung tissues (Dhatwalia et al., 2019), control body weights (Sun et al., 2019) and even come with some antioxidant activities (Zhao et al., 2019). The main volatile compounds in WT were hexaldehyde, (E)‐2‐hexenal, benzaldehyde, phenylacetaldehyde, (E)‐geraniol, phenylethanol, linalool, and linalool oxide (Qi et al., 2018; Wang et al., 2011). Before drinking, WTs are normally cooked at boiling point in a teapot for a short period (Figure 1). To date, researchers have investigated the aroma of WT at room temperatures; however, the aroma of WT in the cooking circumstance had not been elucidated yet. The cooking process might make difference to the volatiles in comparison with dry teas and fresh tea infusions, since the longtime cooking could cause volatiles to evaporate and side reactions could happen under the thermal treatment.

In the present study, SDE was used to extract the volatiles of WT since SDE procedure was similar to tea cooking condition (Figure 1). Furthermore, the SDE extract was analyzed using GC‐MS and GC‐O. This study might help people to understand the aroma characteristic in the circumstance of tea cooking and drinking.

## 2 MATERIALS AND METHODS

### 2.1 Materials

Fifty grams of WT was obtained from Fujian Da Ming Development Company harvested in 2018. Fragrance test strip (160 × 10 × 0.4 mm) was purchased from Guangzhou Zhengmao Printing Co. LTD, the code of fragrance test strip was zmyssxz0901002.

### 2.2 Chemicals

Standard 2‐hexanone, hexanal, 1*H‐*1*‐*ethyl‐pyrrole, 2‐hexenal, *cis*‐3‐hexen‐1‐ol, heptanal, 6‐methyl‐5‐hepten‐2‐one, 2‐pentyl‐furan, 2‐ethyl‐1‐hexanol, benzeneacetaldehyde, *cis‐*linalool oxide, *trans*‐linalool oxide, linalool, 3‐octen‐2‐ol, phenylethyl alcohol, menthol, *α*‐terpineol, safranal, decanal, camphene, geraniol and indole were purchased from Sigma Co. Ltd. Benzyl alcohol, *trans‐β*‐damascenone, *trans‐α*‐ionone, *cis*‐geranylacetone, *trans‐β*‐ionone, 2,4‐ditert‐butylphenol, cedrol, and caryophyllene oxide were purchased from Alfa Aesar Co. Ltd. Standard chemical series of C_8_–C_20_ alkanes that were used to determine the liner retention index (RI) and the internal standard cyclohexanone were obtained from Sigma Co. Ltd. *Cis*‐*pyranoid*‐Linalool oxide and *trans*‐*pyranoid*‐linalool oxide were purchased from Sinopharm Chemical Reagent Co. Ltd.

### 2.3 Preparation of the aroma extract of WT

SDE apparatus was purchased from Beijing Glass Instrument Factory and was similar to the design of Lickens‐Nickerson apparatus. Thirty grams of grounded WT sample was immersed in a 500‐ml flask with 300 ml of distilled water (Chen et al., 2019), and 100 ml of hexane that was applied as extraction solvent was placed in another flask. Both flasks were placed in the Lickens‐Nickerson apparatus heated up to their boiling points. Each extraction was carried out for 1.5 hr after the two flasks started to reflux. After cooling to ambient temperature, the extract was collected and the flask was washed with hexane, which was then combined with the extract. The combined extract was dried over anhydrous sodium sulfate overnight and filtrated. The filtrate was then concentrated approximately to 0.5 ml by using a gentle stream of high‐purity nitrogen and adjusted to the volume of 1.5 ml with hexane. The concentrated extraction was stored at −20°C freezer temporarily before analysis.

### 2.4 Sensory characterization of aroma extract of WT

Four samples were prepared. Original SDE extract (OSDE) were prepared by adjusting 0.5 ml extract to 1.5 ml with hexane. Light white tea infusion (LWTI) was prepared by putting 0.1 g WT in 10 ml of boiling water. Common WT infusion (CWTI) was prepared by putting 0.5 g WT in 10 ml of boiling water. Thick WT infusion (TWTI) was prepared by putting 1.0 g WT in 10 ml of boiling water (Wang, Han, et al., 2019; Wang, Zeng, et al., 2019).

Sensory evaluation was carried out by using the methods of ISO 8,589 and previous study (Chen et al., 2010). Ten panelists, including five women and five men aged between 20 and 30 years old, were trained for 15 hr over a period of 2 weeks to distinguish the aroma descriptions of green, floral, sweet, roasted, and woody. Hexanal (green; Zhang et al., 2018), linalool (floral; Zhang et al., 2018), benzeneacetaldehyde (sweet; Zhu et al., 2017), *trans‐β‐*Ionone (woody; Gong et al., 2017), 2,5‐dimethylpyrazine (roasted; Zhu et al., 2015), and 1‐octanol (fruity; Gong et al., 2017) were used as the standard samples. An aliquot of 20 µl of the WT was diluted with 980 µl of ethanol. After that, 50 µl of the dilution was pipetted onto a fragrance test strip and dried in the open air for 120 s, immediately followed by the sensory evaluation in a clean environment under illumination at 25 ± 2°C using a 9‐point scoring method, in which 1 indicated an unperceived attribute intensity and 9 indicated a very strong attribute intensity. The order of the sensory analysis, green, floral, sweet, roasted, and woody notes were randomly given a score within 1–9. The mean values were calculated after correction of the result and removed the outliners.

### 2.5 GC‐MS analysis

The samples were respectively prepared by mixing 990 µl of the SDE extract with the exact volume of the 10 µl of cyclohexanone which was the internal standard of the samples. After that, each sample was injected into the GC‐MS in 1 µl. A QP2010 GC‐MS (Shimadzu Co., Ltd) and two different fused silica capillary columns, Rtx‐5MS (60 m × 0.32 mm × 0.25 µm; Restek Corporation), and Rtx‐Wax (60 m × 0.32 mm × 0.25 µm; Restek Corporation) were used. The Helium was used as the carrier gas at 3 ml/min. At the same time, the oven temperature initiated from 50ºC for 2 min, then increased to 200ºC in a speed of 3ºC/min and held for 1 min. The temperatures of the ion source and the interface were 220 and 250ºC, respectively. The mass scan range of m/z was set from 35 to 500 amu.

The Kovats method was used to calculate and identify the linear RI using a mixture of n‐alkanes as an external reference. Most of the volatiles were identified by matching their detected MS spectra and Kovats RI to those of standards on both columns and were quantitated according to their respective calibration curves on Rtx‐5MS column using selective ion monitoring mode. Yet some chemicals that lacked standards were tentatively identified based on matching ion fragment and RI values from previous relevant references as well as MS Spectra Library (FFNSC1.3, NIST08, NIST08s) and quantity was analyzed by using the calibration curve of cyclohexanone (internal reference) under the scan mode.

External standard method was used for quantification, the components with standard were quantified by standard curve, the components with internal standard were quantified by cyclohexanone content, and the other components without standard were quantified by peak area comparison.

### 2.6 Gas chromatography‐olfactometry (GC‐MS‐O) analysis

An Agilent 5975C‐7890A GC‐MS (Agilent Technologies) was equipped with an olfactory detection port Gerstel ODP‐2 (Gerstel AG Enterprise). The GC was fitted with HP‐INNOWAX column (60 m × 0.25 mm × 0.25 µm; Agilent). The oven temperature was programmed from 40°C and the initial temperature was 1 min and increased to 230ºC at 5ºC/min and then kept for 3 min, and the high‐purity nitrogen was used as the carrier gas at 1.8 ml/min. The temperature of the injector port was 250°C, and 0.5 µl of each sample was injected into the GC‐MS‐O system. Each GC‐O experiment was evaluated by three panelists (two females and one male). All the panelists were trained for 30 hr over a period of 3 weeks. The aroma character of volatile compounds was evaluated by sniffing, and intensity of the volatile compounds was marked with five scales (“1” mean extremely weak, “3” impress medium odor, “5” mean extremely strong; Sheibani et al., 2016a). These volatile compounds were identified by matching their RI values with standards.

The aroma intensity was based on the aroma character of GC‐O as well as the sensory evaluation.

### 2.7 OAV analysis

To assess the contribution of individual volatiles to the overall aroma distribution, odour activity value(OAV) is one of the important methods to identify compounds by GC‐MS (Tian et al., 2020). The OAV for each volatile was calculated by dividing the concentration of each volatile by their respective thresholds in water (Kesen, 2020). The OAV threshold was taken from available citation. The threshold of safranal was derived from experiments on this reference (Zhu et al., 2017).

### 2.8 Statistical analysis

Samples were analyzed in triplicate. Mean values were calculated using Microsoft Excel 2010 (Microsoft Corporation). The significant analysis was performed by the software SPSS‐IBM 19.0 software (International Business Machines Corporation) and Microsoft Excel 2010 (Microsoft Corporation).

## 3 RESULTS AND DISCUSSION

### 3.1 Sensory evaluation of the SDE extract

It had been reported that WT was an unfermented tea which made by the new growth buds and young leaves (Alcazar et al., 2007). During WT productions, tea leaves were steamed and dried immediately after pluck to avoid oxidation; it had a light and delicate aroma characteristic (Rusak et al., 2008). Perez‐burillo et al. indicated that a WT brewed for 7 min was described mainly by characteristic floral, fruity, and green note (Perez‐Burillo et al., 2018). Qi et al. reported that a fresh WT and a WT in control group were dominated with delicate note and green note; a rapid aged WT was characterized by the strong sweet note, moderate herbal note, and light delicate note; and a natural aged WT was marked by strong herbal note and light sweet note (Qi et al., 2018).

**FIGURE 2:**
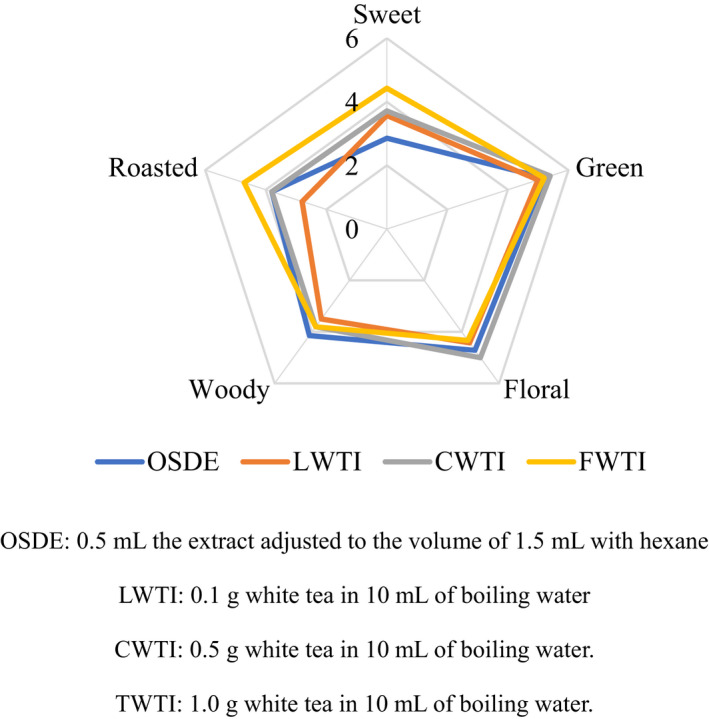
Aroma profiles of the simultaneous distillation‐extraction extract and white tea (WT) infusions. OSDE: 0.5 ml the extract adjusted to the volume of 1.5 ml with hexane. LWTI: 0.1 g WT in 10 ml of boiling water. CWTI: 0.5 g WT in 10 ml of boiling water. TWTI: 1.0 g WT in 10 ml of boiling water

From the Figure 2, it was shown the OSDE was dominated with green, floral, roasted and woody notes, and weak sweet note. LWTI was dominated with green, floral, woody, and sweet notes. Both CWTI and TWTI were dominated with sweet, floral, green woody, and roasted notes, which were different from those of fresh and aged WTs reported by other researchers (Perez‐Burillo et al., 2018; Qi et al., 2018). This difference could be attributed to that the WT used in this experiment was middle aged WT. Although LWTI, CWTI, and TWTI had similar green, floral, and woody notes to OSDE, the sweet note was stronger in those teas than that of OSDE. It indicated the volatiles contributing to sweet note was lost or broken down during the extraction process since researchers have reported that some compounds, especially heat‐sensitive compounds, in tea leaves were apt to change at a high temperature (Gao et al., 2017).

### 3.2 Identification and quantification of volatile compounds in the SDE extract by GC‐MS

An analysis using HS‐SPME‐GC‐GC‐TOFMS showed WT contained 53 aromatic hydrocarbons, 29 esters, 27 alcohols, 26 aldehydes, 18 ketones, 5 heterocyclic compounds, 5 nitrogen compounds, 4 sulfur compounds, 2 phenols, 2 acids, and 1 ether (Chen et al., 2019). Wang et al. reported that the main volatile compounds in a WT were hexanal, (E)‐2‐hexenal, benzaldehyde, benzeneacetaldehyde, (E)‐geraniol, phenylethyl alcohol, linalool, and linalool oxide (Wang et al., 2011). Qi et al. reported there were 30 substances in a rapidly aged WT via HS‐SPME analysis, including 4 alcohols, 5 aldehydes, 9 ketones, 5 esters, 4 alkenes, and 3 others (Qi et al., 2018).

In this study, thirty‐two volatile compounds were detected on Rtx‐5ms and Rtx‐wax columns according to their RI, mass spectrum, and characteristic fragments. These volatiles included 12 alcohols, 6 aldehydes, 6 ketones, 5 oxides, and 3 others (Table 1). Among them, 29 volatile compounds were quantitively analyzed using their respective standard curves, and the other 3 compounds were estimated by the internal standard method due to lack of standards (Table 1). In the SDE extract, the main alcohols were menthol (84.4 mg/L) and benzyl alcohol (35.6 mg/L); aldehydes were mainly benzeneacetaldehyde (19.8 mg/L), 2‐hexenal (11.7 mg/L), and hexanal (9.9 mg/L); ketones were mainly 2‐hexanone (16.9 mg/L); oxides were mainly *trans*‐pyranoid‐linalool oxide (18.2 mg/L), *cis*‐pyranoid‐linalool oxide (9.9 mg/L), *trans*‐linalool oxide (9.3 mg/L), and *cis*‐linalool oxide (8.2 mg/L; Table 2). Comparing with relevant articles (Chen et al., 2019; Qi et al., 2018), more menthol (84.3 mg/L), cedrol (6.6 mg/L), decanal (7.3 mg/L), and indoles (1.3 mg/L) were detected in the SDE extract, indicating a noticeable amount of menthol, cedrol, decanal, and indoles were generated in the SDE extraction. This result was similar to the studies that the black tea under high steeping temperature conditions had higher aroma‐active components than that in the counterpart under common temperature (Wang, Han, et al., 2019; Wang, Zeng, et al., 2019). On the other hand, esters were hardly to be detected in the SDE extract. Wu et al. reported that the high‐temperature filming of green tea may lead to the loss of some ester compounds (Wu et al., 2016). Therefore, it is concluded that thermal treatment during SDE extract and WT cooking lead to the promotion of alcohols with high boiling points and the loss of esters with low boiling points.

**TABLE 1:** Identification, standard curves and concentrations of the volatile compounds of the simultaneous distillation‐extraction extract

No	RTX−5MX		RTX‐WAX		Reference^e^	Volatile	Standard curve^f^	*R* ^2^	CI	Range (mg/L)	Concentration (mg/L)	CF^g^
	RI^a^	RI^b^	RI^c^	RI^d^								
1	—	792	—	1,098	Std MS	2‐Hexanone	*Y* = 0.2404*X* − 0.2621	0.9990	43 58 57	0.5–100	16.9 ± 0.7	0.264
2	800	800	1,075	1,078	Std MS R1	Hexanal	*Y* = 1.3054*X* − 0.0609	0.9997	44 41 56	0.5–100	9.9 ± 0.7	0.771
3	849	850	—	1,216	Std MS R1	2‐Hexenal	*Y* = 0.5910*X* − 0.0432	0.9997	41 69 42	0.5–100	11.7 ± 0.5	1.270
4	854	855	1,388	1,386	Std MS R1	*cis‐*3‐Hexen*‐*1‐ol	*Y* = 1.6069*X* − 0.0631	0.9998	41 67 82	0.5–100	3.7 ± 0.2	0.625
5	902	902	1,189	1,188	Std MS R1	Heptanal	*Y* = 1.5103*X* − 0.0835	0.9997	44 70 43	0.5–100	1.8 ± 0.1	0.667
6	988	988	1,383	1,387	Std MS R1	*6*‐methyl*‐*5‐Hepten*‐*2‐one	*Y* = 2.6180*X* − 0.1443	0.9997	43 41 108	0.5–100	1.0 ± 0.1	0.385
7	992	991	1,227	1,229	Std MS R1	*2*‐pentyl‐Furan	*Y* = 3.3982*X* − 0.1975	0.9995	81 82 138	0.5–100	1.6 ± 0.0	0.296
8	1,030	1,030	—	1,484	Std MS R1	2‐ethyl*‐*1‐Hexanol	*Y* = 3.3049*X* − 0.0446	0.9998	57 41 43	0.5–100	0.7 ± 0.0	0.302
9	1,036	1,037	1,874	1,877	Std MS R1	Benzyl alcohol	*Y* = 0.1963*X* − 0.4311	0.9970	108 79 107	0.5–100	35.6 ± 5.3	0.541
10	1,044	1,044	1,628	1,626	Std MS R1	Benzeneacetaldehyde	*Y* = 1.3974*X* − 0.0543	0.9995	91 92 120	0.5–100	19.8 ± 1.5	0.719
11	1,072	1,073	1,439	1,438	Std MS R1	*cis*‐Linalool oxide	*Y* = 2.8420*X* − 0.0683	0.9997	59 43 94	0.5–100	8.2 ± 1.1	0.354
12	1,088	1,088	1,468	1,471	Std MS R1	*trans*‐Linalool oxide	*Y* = 4.3092*X* − 0.0727	0.9997	59 94 43	0.5–100	9.3 ± 0.2	0.233
13	1,100	1,100	1,552	1,552	Std MS R1	Linalool	*Y* = 2.2918*X* − 0.1747	0.9996	71 41 93	0.5–100	5.5 ± 0.1	0.441
14	1,105	1,107	—	1,623	MS R2	Hotrienol					6.8 ± 0.3	
15	1,108	1,042	1,590	—	Std MS R3	3‐Octen*‐*2‐ol	*Y* = 3.6322*X* − 0.2084	0.9991	81 39 110	0.5–100	2.4 ± 0.1	0.277
16	1,114	1,114	1,907	1,912	Std MS R1	Phenylethyl alcohol	*Y* = 7.6178*X* − 0.5345	0.9995	91 92 122	0.5–100	2.2 ± 0.5	0.132
17	1,170	1,167	1,738	1,742	MS R1	*cis*‐*pyranoid*‐Linalool oxide					9.9 ± 3.1	
18	1,174	1,173	1,595	1,608	Std MS R1	Menthol	*Y* = 0.2444*X* − 0.0135	0.9996	71 81 95	0.5–100	84.4 ± 2.2	4.121
19	1,175	1,173	1,767	1,759	MS R1	*trans*‐*pyranoid*‐Linalool oxide					18.2 ± 0.8	
20	1,192	1,190	1,695	1,692	Std MS R1	*α*‐Terpineol	*Y* = 1.2469*X* − 0.0785	0.9994	59 93 121	0.5–100	2.4 ± 0.2	0.808
21	1,201	1,201	1,631	1,648	Std MS R1	Safranal	*Y* = 2.6516*X* − 0.0499	0.9998	107 91 121	0.5–100	1.0 ± 0.1	0.378
22	1,206	1,207	1,494	1,495	Std MS R1	Decanal	*Y* = 0.3174*X* − 0.0331	0.9992	43 41 57	0.5–100	7.3 ± 2.1	3.192
23	1,230	—	—	1,059	Std MS R1	Camphene	*Y* = 0.2809*X* + 0.0015	0.9999	93 121 79	0.5–100	1.5 ± 0.1	3.550
24	1,259	1,260	1,854	1,854	Std MS R1	Geraniol	*Y* = 1.6663*X* − 0.1813	0.9994	69 41 68	0.5–100	1.4 ± 0.00	0.603
25	1,299	1,295	2,345	2,414	Std MS R1	Indole	*Y* = 4.5914*X* − 0.4733	0.9994	117 90 89	0.5–100	1.3 ± 0.0	0.221
26	1,387	1,386	1,810	1,810	Std MS R1	*trans‐β‐*Damascenone	*Y* = 1.3702*X* − 0.0799	0.9998	177 69 41	0.5–100	1,8 ± 0.1	0.735
27	1,431	1,429	1,841	1,844	Std MS R1	*trans‐α*‐Ionone	*Y* = 3.1402*X* − 0.1273	0.9999	121 93 43	0.5–100	1.4 ± 0.0	0.320
28	1,455	1,457	—	1,835	Std MS R1	*cis*‐Geranylacetone	*Y* = 2.3970*X* − 0.1372	0.9998	44 69 41	0.5–100	2.4 ± 0.8	0.420
29	1,490	1,490	1,930	1,926	Std MS R1	*trans‐β*‐Ionone	*Y* = 7.7843*X* − 0.1273	0.9995	177 43 41	0.5–100	2.3 ± 0.0	0.129
30	1,516	1,513	—	2,321	Std MS R1	2,4‐Ditert‐butylphenol	*Y* = 17.5469*X* + 0.1502	0.9998	191 57 206	0.5–100	0.2 ± 0.0	0.057
31	1,608	1,608	—	2,112	Std MS R1	Cedrol	*Y* = 1.1834*X* − 0.0940	0.9995	95 150 151	0.5–100	6.6 ± 0.4	0.867
32	1,615	1,613	—	2,014	MS R1	Caryophyllene oxide	*Y* = 3.7303*X* − 0.3035	0.9995	43 41 79	0.5–100	1.0 ± 0.0	0.271

^a^ Retention index (RI) is obtained by gas chromatography–mass spectrometry (GC‐MS) analysis using the Rtx‐5MS column.

^b^ RI is reported in the literature or websites and is analyzed using a column similar to Rtx‐5MS.

^c^ RI is obtained by GC‐MS analysis using the Rtx‐wax column.

^d^ RI is reported in the literature or websites using a column similar to Rtx‐wax.

^e^ Std indicates that the identification was confirmed by matching a standard, and the number following R is the corresponding reference showing the RI values (R1 is referred to the database on the web (http://webbook.nist.gov/chemistry/—R2 is Zhang et al., 2018).

^f^ All of the equations of the calibration curves of authentic standard chemicals (ASCs) are calculated in the selective ion monitoring (SIM) mode, where *X* is the ratio of the concentration of the ASC to that of the internal standard (IS) and *Y* is the ratio of the peak area of the ASC to that of the IS.

^g^ CF represents correction factors using this formula: CF = (As/Ms)/(Ar/Mr), As represents the corresponding quantitative ion (SIM mode) area of the IS, Ar is the corresponding quantitative ion (SIM mode) area of the ASC, Ms is the concentration of IS, and Mr represents the concentration of the ASC.

**TABLE 2:** Gas chromatography‐olfactometry analysis of aroma descriptions of the volatiles in the simultaneous distillation‐extraction extract

No.	RI	Volatiles	Aroma description	Aroma intensity
1	1,072	2‐Hexaone	Floral, sweet	2.3
2	1,388	Hexanal	Green	3.0
3	1,225	2‐Hexenal	Green	1.7
4	1,371	*cis‐*3‐Hexen*‐*1‐ol	Fruit, green	1.3
5	1,223	2‐Pentyl‐furan	Green, roasted	4.7
6	1,876	Benzyl alcohol	Sweet	1.7
7	1,655	Benzeneacetaldehyde	Sweet, floral	3.0
8	1,442	*cis*‐Linalool oxide	Floral	1.7
9	1,460	*trans*‐Linalool oxide	Floral	3.0
10	1,535	Linalool	Floral	4.7
11	1,599	Hotrienol	Green, floral	1.3
12	1,636	Menthol	Minty	4.3
13	1,735	*α*‐Terpineol	Green, wood	‐
14	1,687	Safranal	Herbal	2.0
15	1,075	Camphene	Green	3.3
16	1,859	Geraniol	Sweet, floral	1.3
17	1,832	*trans‐β*‐Damascenone	Sweet	4.7
18	1,843	*trans‐α*‐Ionone	Wood, stale	2.0
19	1,955	*trans‐β*‐Ionone	Sweet, wood	2.3

### 3.3 Investigation of the aroma descriptions of volatile compounds by GC‐O

Hexanal and 2‐hexenal were related to a green and grassy notes in black tea leaves (Wang et al., 2011). Menthol with mint note was one of the most important aroma‐active compound of raw Pu‐erh (Xu et al., 2016). *cis*‐3‐Hexen‐1‐ol was related to the green note in Oolong tea infusion (Zhu et al., 2015). *cis*‐Linalool oxide, *trans*‐linalool oxide, geraniol, and *trans‐β‐*ionone were related to the floral odor in Oolong tea infusion (Zhu et al., 2015). Benzeneacetaldehyde and *β*‐damascenone were important contributors to the sweet note in Oolong tea infusion (Zhu et al., 2015). Linalool was identified as the contributor to citrus and floral notes of teas (Schuh & Schieberle, 2006). Safranal was described possessing sweet, green, and floral notes in Kangra orthodox black tea (Joshi & Gulati, 2014). In Pu‐erh tea, 2‐pentyl‐furan and benzyl alcohol presented fruity note; hotrienol presented stale note; *α‐*terpineol presented sweet and green notes; *trans‐α‐*ionone presented woody note (Xu et al., 2016). For the Chinese congou black tea, 1‐penten‐3‐ol, *cis*‐6‐nonen‐1‐ol, 2,3‐butanedione, 2‐heptanone, 2‐methylpyrazine, and 2‐ethyl‐5‐methyl‐pyrazine showed positive contribution to the roasted note (Chen et al., 2019); hexanol, (E)‐2‐hexen‐1‐ol, 2‐furanmethanol, *cis*‐6‐nonen‐1‐ol, hexanal, furfural, methyl salicylate, 2,3‐butanedione, cis‐jasmone, 2‐methylpyrazine and 2‐ethyl‐5‐methyl‐pyrazine showed a positive influence on the sweet note (Chen et al., 2019); *cis*‐linalool oxide, trans‐linalool oxide, and nerolidol showed positive correlation with the woody note (Xiao et al., 2017). It was obvious that different types of teas have different aroma‐active volatile compounds.

For the SDE extract, a total of 19 volatiles showed noticeable green, floral, sweet, woody and roasted notes based on the GC‐O analysis (Table 2). Among these, hexanal(3.0), 2‐hexenal(1.7), *cis*‐3‐hexen‐1‐ol(1.3), 2‐pentyl‐furan(4.7), hotrienol(1.3), *α*‐terpineol and camphene(3.3) presented green note; 2‐hexanone (2.3), *trans*‐linalool oxide (3.0), benzeneacetaldehyde (3.0), linalool (4.7), *cis*‐linalool oxide (1.7), hotrienol(1.3), and geraniol (1.3) presented floral note; 2‐hexanone (2.3), geraniol (1.3), benzyl alcohol (1.7), *trans‐β*‐damascenone (4.7), and *trans‐β*‐ionone (2.3) presented sweet note; *trans‐α*‐ionone (2.0) and *trans‐β*‐ionone (2.3) presented woody note; 2‐pentyl‐furan (4.7) presented roasted note; safranal (2.0) had a positive influence on herbal note; and menthol (4.3) had a positive influence on minty note. Qi et al. reported that *cis*‐geraniol, phenylacetaldehyde, 5‐methyl‐2‐furaldehyde, *β*‐damascenone, and methyl hexanoate presented sweet note in aged WT (Qi et al., 2018), which was different from the results of this study that 2‐hexanone, geraniol, *trans‐β*‐damascenone, and *trans‐β*‐ionone contributed to sweet note; the difference of the two results may be related to that 5‐methyl‐2‐furaldehyde and methyl hexanoate evaporate at high temperature.

### 3.4 Investigation of aroma‐active volatile compounds by OAV analysis

In order to clarify the influence of candidate chemicals on aroma characteristics, OAVs of 19 volatiles were estimated according to their concentrations and thresholds. OAV value greater than 1 was regard as to have a marked effect on aroma(Li et al., 2018). As shown in Table 3, for OSDE, the OAVs for hexanal (OAV 2,209.9), 2‐hexenal (OAV 143.3), *cis*‐3‐hexen‐1‐ol (OAV 52.4), 2‐pentyl‐furan (OAV 265.5), benzyl alcohol (OAV 3.6), benzeneacetaldehyde (OAV 4,949.7), *cis*‐linalool oxide (OAV 25.5), *trans*‐linalool oxide (OAV 29.2), linalool (OAV 919.9), hotrienol (OAV 61.5), *α*‐terpineol (OAV 7.4), safranal (OAV 1,453.7), geraniol (OAV 34.0), *trans‐β*‐damascenone (OAV 1,398,769.0), *trans‐α*‐ionone (OAV 362.2), and *trans‐β*‐ionone (OAV 335,285.7) was greater than 1. Furthermore, hexanal (OAV 2,209.9), 2‐hexenal (OAV 143.3), and *cis*‐3‐hexen‐1‐ol (OAV 52.4) contributed the majority of OAVs of green note; 2‐pentyl‐furan (OAV 265.5) and benzyl alcohol (OAV 3.6) contributed the majority of OAVs of roasted note; benzeneacetaldehyde (OAV 4,949.7), *cis*‐linalool oxide (OAV 25.5), *trans*‐linalool oxide (OAV 29.2), geraniol (OAV 34.0), and linalool (OAV 919.9) contributed the majority of OAVs of floral note; *trans‐α*‐ionone (OAV 362.2), *trans‐β*‐ionone (OAV 335,285.7), and *trans*‐linalool oxide (OAV 29.2) contributed the majority of OAVs of woody note; benzyl alcohol (OAV 3.6), benzeneacetaldehyde (OAV 4,949.7), and *trans‐β*‐damascenone (OAV 1,398,769.0) contributed the majority of OAVs of sweet note.

**TABLE 3:** OAV analysis of the aroma‐active volatiles in the simultaneous distillation‐extraction extract

No.	Volatiles	Aroma description	Threshold value (μg/L)	Concentration (mg/L)	OAV
1	Hexanal	Green	4.5 (Zhu et al., 2015)	9.9 ± 0.7	2,209.9
2	2‐Hexenal	Green	82 (Zhu et al., 2015)	11.7 ± 0.5	143.3
3	*cis‐*3‐Hexen*‐*1‐ol	Green	70 (Zhu et al., 2015)	3.7 ± 0.2	52.4
4	2‐Pentyl‐furan	Roasted	5.9 (Zhu et al., 2015)	1.6 ± 0.0	265.5
5	Benzyl alcohol	Roasted, sweet	10,000 (Zhu et al., 2015)	35.6 ± 5.3	3.6
6	Benzeneacetaldehyde	Sweet, floral	4 (Li et al., 2018)	19.8 ± 1.5	4,949.7
7	*cis*‐Linalool oxide	Floral	320 (Zhu et al., 2015)	8.2 ± 1.1	25.5
8	*trans*‐Linalool oxide	Floral, woody	320 (Zhu et al., 2015)	9.3 ± 0.2	29.2
9	Linalool	Floral	6 (Li et al., 2018)	5.5 ± 0.1	919.9
10	Hotrienol	Floral	110 (Zhu et al., 2015)	6.8 ± 0.3	61.5
11	*α*‐Terpineol	Woody	330 (Zhu et al., 2015)	2.4 ± 0.2	7.4
12	Safranal	Herbal	0.7	1.0 ± 0.1	1,453.7
13	Geraniol	Floral	40 (Zhu et al., 2015)	1.4 ± 0.0	34.0
14	*trans‐β*‐Damascenone	Sweet	0.0013 (Zhu et al., 2015)	1.8 ± 0.1	1,398,769.0
15	*trans‐α*‐Ionone	Woody	4 (Li et al., 2018)	1.4 ± 0.0	362.2
16	*trans‐β*‐Ionone	Woody	0.007 (Li et al., 2018)	2.3 ± 0.0	335,285.7

It has been reported that the aroma components in WT belonged to endogenous biosynthetic volatiles, including fatty acid volatiles, amino acid volatiles, terpenoid volatile, and carotenoid volatiles (Chen et al., 2019). The biosynthesis of monoterpenes and sesquiterpenes was usually carried out by cytosol‐localized mevalonate and plastid‐localized methylerythritol phosphate pathways in plants (Chen et al., 2019). Hexanal and 2‐hexanal were metabolized from unsaturated long‐chain fatty acids, such as *α‐*linolenic acid and linoleic acid, through the lipoxygenase enzymatic pathway to produce lipid hydroperoxides, then cleaved by fatty acid hydroperoxide lyase into six carbon fat volatile compounds (Chen et al., 2019; Hu et al., 2018). Phenylacetaldehyde may be derived from the oxidative degradation of the amino acid phenylalanine (Wang et al., 2017), phenylalanine was transformed to phenylacetaldehyde by deamination, and benzaldehyde and benzyl alcohol were derived from phenylpyruvic acid (Wang, Han, et al., 2019; Wang, Zeng, et al., 2019). *β*‐Ionone was the product of oxidative degradation of carotenoids and can be increased by thermal degradation (Gungr et al., 2018). It was reported that heat treatment can destroy indole precursor and lead to the change of indole content in teas (Sheibani et al., 2016). (Z)‐3‐Hexene‐1‐ol was transformed from (Z)‐3‐hexenyl glycosides (Wang, Han, et al., 2019; Wang, Zeng, et al., 2019). Linalool and geraniol were produced by hydrolysis of nonvolatile *β‐D‐*glycosides, nonenzymatic hydrolysis of glycolate‐volatile compounds occurred during the tea‐producing process, and the hydrolysis were increased at high temperature, and linalool oxide were synthesized from linalool (Gungr et al., 2018; Wang et al., 2016). Under heating conditions, 2‐pentyl‐furan may be produced by Maillard reaction (Mao et al., 2018). Alcohols, aldehydes, and ketones were formed by the degradation of carotenoids, the oxidation of unsaturated fatty acids, or the hydrolysis of glycosides; these reactions usually related to endogenous enzymes (Tan et al., 2019). In tea leaves, volatile compounds of tea occurred in both free and glycosidically bound forms (Zeng et al., 2019).

**FIGURE 3:**
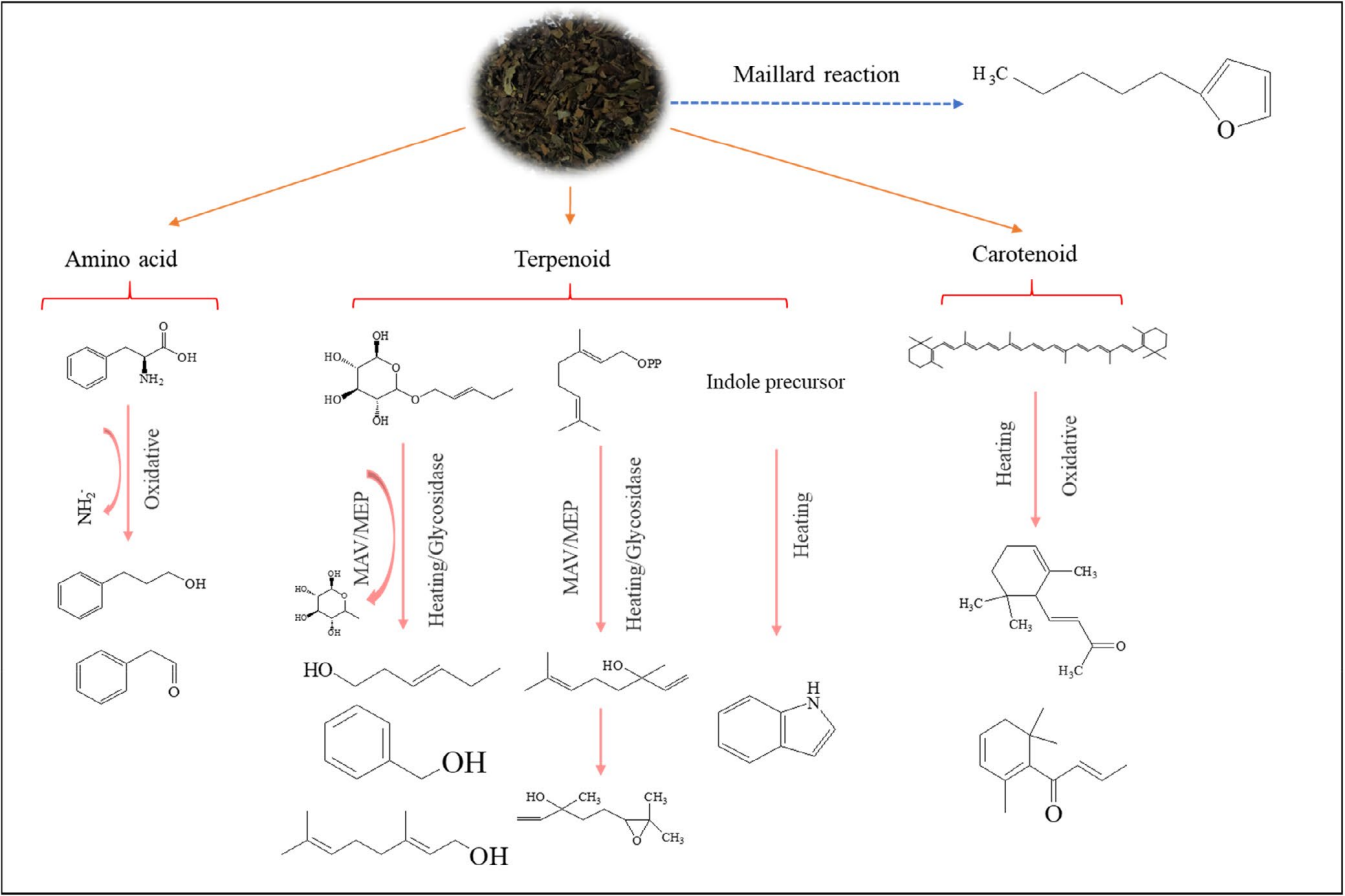
The synthesis pathway of volatile components of the simultaneous distillation‐extraction extract

Based on the above analysis and literatures, the putative reaction pathways could be divided into four branches to explain aromatic‐active volatiles (Figure 3), including oxidation of amino acid, degradation of carotene, Maillard reaction, and hydrolysis of glycoside compounds at high temperatures. Phenylacetaldehyde may be derived from the oxidative degradation of the amino acid phenylalanine (Wang et al., 2017). *β‐*Carotene degrade to generate *β‐*ionone and derived to form *β‐*damascenone at the high temperatures (Gao et al., 2017; Tan et al., 2019). Maillard reactions were apt to occur in case of heating, producing heterocyclic compounds such as furan, pyrrole, thiophene, and their derivatives (Mao et al., 2018), which might explain the generation of 2‐pentyl‐furan. Geraniol, linalool, linalool oxide, and benzyl alcohol may be produced from glycosides via thermal hydrolysis (Mao et al., 2018; Wang, Han, et al., 2019; Wang, Zeng, et al., 2019) revealing the causing of high content of alcohols in SDE extract. The results enriched our knowledge on tea aroma under cooking as well as other thermal treatments. In the future, the aroma components are expected to be dynamically studied on molecular reaction mechanism using isotope labeling experiment.

## 4 CONCLUSIONS

The SDE extract and the WT had similar intensities for floral, sweet, green, woody, and roasted notes, whereas the SDE extract had a weaker sweet note than the WT without cooking. Hexanal, 2‐hexenal, *cis*‐3‐hexen‐1‐ol, and camphene were the main contributors to the green note. Benzeneacetaldehyde, 2‐hexanone, *trans*‐linalool oxide, and linalool were the main contributors to the floral note. *trans*‐*β*‐Damascenone was the main contributors to the sweet note. 2‐Pentyl‐furan was the main contributors to the roasted note. *trans‐α*‐Ionone and *trans‐β*‐ionone were the main contributors to the woody note. The aromatic‐active volatiles generated at the SDE and tea cooking circumstance were related in four putative reaction pathways, including amino acid degradation, carotene degradation, Maillard reaction, and glycosides hydrolysis.

## CONFLICT OF INTEREST

The authors declare that they do not have any conflict of interest.

## AUTHOR CONTRIBUTIONS

Qi Lin: Data curation, Writing—original draft; Hui Ni: Writing review and editing, Conceptualization; Ling Wu: Visualization, Investigation; Shu Yi Weng: Resources; Li Jun Li: Project administration, Validation; Feng Chen: Supervision.

## ETHICAL REVIEW

This study does not involve any human or animal testing.

## INFORMED CONSENT

Written informed consent was obtained from all study participants.

## Data Availability

Research data are not shared.
